# Design and Fabrication of High-Performance Piezoelectric Micromachined Ultrasonic Transducers Based on Aluminum Nitride Thin Films

**DOI:** 10.3390/mi15081001

**Published:** 2024-08-01

**Authors:** Le Zhang, Kunxian Yan, Lei Ye, Xiangyu Luo, Jian He, Xiujian Chou

**Affiliations:** Science and Technology on Electronic Test and Measurement Laboratory, North University of China, Taiyuan 030051, China; kunxian_yan1997@163.com (K.Y.); ye951107797@gmail.com (L.Y.); sz202206022@st.nuc.edu.cn (X.L.); drhejian@nuc.edu.cn (J.H.); chouxiujian@nuc.edu.cn (X.C.)

**Keywords:** piezoelectric micromachined ultrasonic transducer (PMUT), aluminum nitride (AlN), microelectromechanical system (MEMS) fabrication, FEM

## Abstract

Ultrasound is widely applied in diverse domains, such as medical imaging, non-destructive evaluation, and acoustic communication. Piezoelectric micromachined ultrasonic transducers (PMUTs) capable of generating and receiving ultrasonic signals at the micrometer level have become a prominent technology in the field of ultrasound. It is important to enrich the models of the PMUTs to meet the varied applications. In this study, a series of PMUT devices featured with various top electrode configurations, square, circular, and doughnut, were designed to assess the influence of shape on the emission efficacy. It was demonstrated that the PMUTs with a circular top electrode were outperformed, which was calculated from the external acoustic pressure produced by the PMUTs operating in the fundamental resonant mode at a specified distance. Furthermore, the superior performance of PMUT arrays were exhibited through computational simulations for the circular top electrode geometries. Conventional microelectromechanical systems (MEMS) techniques were used to fabricate an array of PMUTs based on aluminum nitride (AlN) films. These findings make great contributions for enhancing the signal transmission sensitivity and bandwidth of PMUTs, which have significant potential in non-destructive testing and medical imaging applications.

## 1. Introduction

Ultrasound represents a category of sound waves characterized by frequencies exceeding the audible range for humans [1], typically surpassing 20 kilohertz (kHz). Owing to the elevated frequency spectrum, ultrasound possesses distinctive attributes, including non-destructiveness, heightened sensitivity, and inherent safety [2]. Ultrasonic transducers, serving as both transducers and actuators, are employed across a variety of application scenarios within the ultrasonic frequency range from 20 kHz to 40 MHz, which is due to the diverse ultrasonic properties exhibited at different frequencies [3]. For example, ultrasound with higher frequencies offers superior resolution and is extensively utilized in the medical sector because it is safe and non-invasive, so it is chosen the patients and also preferred by physicians when medical imaging is needed. An ultrasonic transducer is an apparatus that converts electrical signals into sonic waves. And it can be categorized into piezoelectric or capacitive transducers, depending on the unique operating principles and modes. Among these, piezoelectric micro ultrasound transducers [4,5,6] represent a revolutionary category of microelectromechanical system (MEMS) [7,8] devices. They have attracted considerable interest in various ultrasound fields due to the primary advantages of PMUTs, including the compact size, adaptable design, and outstanding performance capabilities, which collectively present a broad spectrum of potential applications.

The PMUT leverages the piezoelectric effect of piezoelectric materials to facilitate the conversion between electrical and acoustic signals, which are sound waves that propagate through a medium. Given that sound waves are mechanical in nature, they induce vibrations in thin film at the same frequency, but varying amplitudes. Owing to the positive piezoelectric effect, when PMUTs are subjected to mechanical stress, an electric charge is generated on their surface, which enables the piezoelectric film layer to transform the vibration of an acoustic wave into a corresponding charge signal. To gather the charges produced by the piezoelectric membrane layer, it is vital to design the structure of PMUT electrodes reasonably well. These electrodes efficiently collect the charge signals, achieving the transformation of ultrasonic waves into electrical signals [9]. Nevertheless, PMUTs’ functionality extends beyond converting sound waves into electrical signals; they can also perform the reverse process, that is, converting electrical signals into sound waves, which can be considered as the inverse process in the ultrasonic transmission mode. Initially, an electrical signal at a specific frequency is applied across the piezoelectric membrane material. Due to the inverse piezoelectric effect, the piezoelectric film material deforms at the same frequency as the electrical signal. This mechanical deformation induces a pressure difference in the medium and propagates outward, resulting in the creation of sound waves. Through this mechanism, the PMUT achieves the capability to transmit ultrasonic waves [10,11]. This capability enables the PMUT to be utilized in a diverse range of applications across various fields. In essence, the PMUT realizes efficient interconversion between the electrical and acoustic signals by leveraging the piezoelectric and inverse piezoelectric effects of piezoelectric thin-film materials for a multitude of applications [12,13].

Aluminum nitride (AlN) is a widely studied and utilized material for the fabrication of PMUTs [14,15,16,17]. AlN is a potential piezoelectric material with numerous advantages. Firstly, according to the calculation formula, the higher piezoelectric constant of AlN guarantees higher sensitivity. Secondly, AlN has a relatively high elasticity constant, allowing it to withstand greater pressure without fracturing. Moreover, compared to other materials, AlN exhibits superior temperature stability, enabling it to maintain good equipment performance under a variety of environmental conditions. These exceptional properties make AlN an ideal choice for PMUT manufacturing. Unlike Capacitive Micro Ultrasonic Transducers (CMUTs) with a cavity structure [18,19], which require a higher DC voltage bias to achieve a balance, AlN-PMUTs could be derived by only a low alternating voltage, which make fabrication easy and are energy efficient. Furthermore, due to the inherent compatibility between AlN and CMOS processes [20], AlN-PMUTs on CMOS-based circuits with a high-bandwidth ultrasound signal processing capability are easily fabricated without the need for complex interface circuits [21]. Due to these outstanding characteristics, PMUTs using AlN as a piezoelectric material hold great promise for a wide range of applications in the ultrasound fields of medical ultrasound imaging.

In the realm of medical imaging, the performance metrics, including sensitivity, resolution, and signal-to-noise ratio [7,22], were closely related with the design and fabrication of PMUTs. In general, high-frequency transducers operated within a frequency band exceeding 5 MHz is beneficial to obtain to a higher resolution [23]. With technological advancements, conventional cart-based ultrasound equipment is progressively being supplanted by handheld devices. These units are compact and portable, offering versatile imaging capabilities for various body parts. To meet the typical requirements of medical ultrasound, the high performance of broader bandwidths of PMUTs is badly needed to enhance the output pressure in an efficient way. Simultaneously, PMUTs must be seamlessly integrated with other devices to ensure compatibility and stability, thereby optimizing their contributions to medical imaging.

In this study, PMUTs with a variety of upper electrode configurations were meticulously designed and fabricated. The performance disparities observed in PMUT cell and arrays with diverse electrode geometries were meticulously evaluated using the Finite Element Method (FEM) to discern the repercussions of the variances on the efficacy of medical imaging. A comprehensive, geometrically integrated 3D FEM model was developed, characterized by a traditional sandwich structure. The modeling process was predicated on periodic boundary constraints. To authentically replicate the influence of PMUTs when applied to human tissue, the electroacoustic performance of the PMUT arrays in aqueous environments were emulated employing a multiphysics field coupling technique [13]. This methodological approach simulates the process of ultrasound in aquatic conditions as that in the human body, which could mitigate the discrepancies between the simulation results and actual scenarios. Through theoretical analysis coupled with multi-physics field simulation of PMUTs, the superior performance of PMUT arrays were samples with circular top electrode geometries. Furthermore, the impact of thin-film vibrating layer structural parameters on the eigenfrequency of the PMUTs was examined through parameter scanning of the established PMUT model parameters. At the end, conventional MEMS techniques were used to fabricate an array of PMUTs based on AlN films using an SOI substrate.

## 2. PMUT Modeling and Simulation

### 2.1. Design of PMUT

The design process for PMUTs involves several key steps. Firstly, the material selection for the ultrasonic transducer is determined. Next, the PMUT unit parameters are determined based on the predetermined operating frequency, and a simulation model is established accordingly. Finally, the top electrode pattern with the best performance is selected to form the top electrode of the PMUT array, and the performance parameters of the PMUT array are explored in this manner [24]. This iterative process ensures that the final PMUT design meets the desired specifications for medical imaging applications.

**Figure 1:**
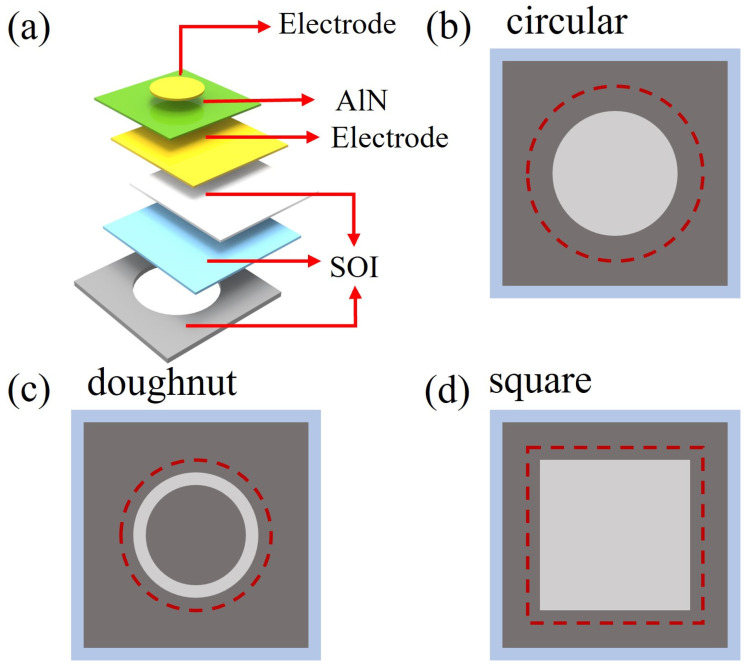
(**a**) Schematic of PMUT unfolding. (**b**) Circular top electrode. (**c**) Doughnut top electrode. (**d**) Square top electrode.

The 3D unfolding of the PMUT is schematically represented in Figure 1, with a circular top electrode pattern used as an example. The top view layout is shown in Figure 1b–d; the bottom cavity and upper electrode dimensions of all the three models are to be kept within the appropriate size range to eliminate the influence of objective factors such as cavity size. The piezoelectric layer materials chosen for the design of PMUTs are typically lead zirconate titanate (PZT) and AlN. Aluminum nitride has excellent thermal conductivity and electrical insulation properties, while the piezoelectric coefficient is relatively small, making it easier to integrate with CMOS in MEMS fabrication. A comparison of the parameters of PZT and AlN is provided in Table 1 below.

The transmit sensitivity $S_{e}$ and receive sensitivity $S_{R}$ of a piezoelectric ultrasound transducer can be defined as follows [25]:

(1) $$S_{e}\propto e_{31f}$$

(2) $$S_{R}\propto e_{31f}/\varepsilon_{33}$$

where $e_{31f}$ is the effective piezoelectric constant, and $\varepsilon_{33}$ is the dielectric constant. The comparative relationship between the piezoelectric materials, PZT and AlN, is shown in Table 1. It was calculated that the emission performance of PZT is better than that of AlN. However, AlN has a stronger receiving ultrasonic performance and higher safety, making it a more suitable choice for the piezoelectric layer material in the field of ultrasonic imaging [26]. Therefore, AlN is selected as the piezoelectric layer material for the design of the PMUTs in this study.

### 2.2. Modelling of PMUT

**Figure 2:**
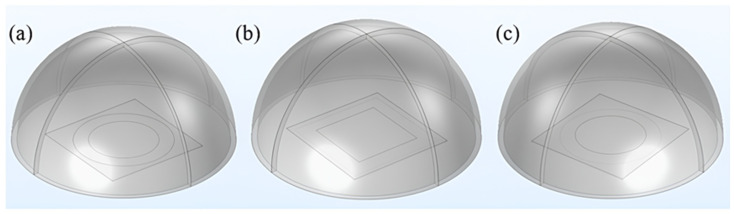
(**a**) Doughnut model. (**b**) Square model. (**c**) Circle model.

In this study, the performance parameters of three aluminum nitride-based PMUT cells and the corresponding arrays were analyzed using a three-dimensional modeling approach. As shown in Figure 2, three PMUT cells with different electrode patterns and back cavity shapes were constructed using COMSOL Multiphysics 6.0. In this paper, the side length of the square upper electrode and the diameter of the circular upper electrode are equal to 252 μm, the side length of the square back cavity is 360 μm, and the diameter of the circular back cavity is 360 μm. The inner and outer diameters of the ring upper elec-trode are 252 μm and 360 μm, respectively, and the diameter of the ring back cavity is 360 μm. Information about the related materials can be found in the COMSOL simulation material library.

In the process of modeling the PMUT array, fixed boundary constraints are applied to the array. Since ultrasound propagation in the human body can be compared to that in water, the external physical field sound propagation domain is set to be the water domain. The mesh profile is chosen as one-fifth of the length of the ultrasound waves in the water domain. When the PMUT array is excited by a given alternating voltage, multiple excited modes will be generated in the simulation results. However, higher-order modes are difficult to be excited in practice, and the working state is unstable. Therefore, the first-order resonance mode (0, 1) is selected to focus on the acoustic characteristics of the PMUT arrays.

## 3. Fabrication

**Figure 3:**
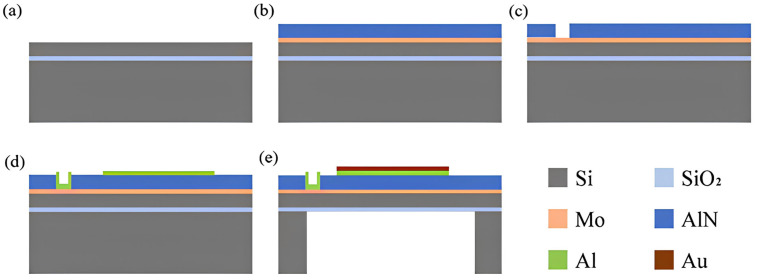
(**a**) SOI wafer. (**b**) AlN deposition on the upper surface. (**c**) Etch AlN until the lower electrode Mo leaks out. (**d**) Deposition of electrode Al. (**e**) Deposit Au and cover the upper electrode.

The PMUTs are fabricated using micro-nano-machining technology, as shown in the manufacturing process depicted in Figure 3.

The substrate is a 4-inch SOI wafer. The SOI wafer consists of a 5 μm top silicon layer, a 400 μm bottom silicon layer, and a 1 μm oxide buried layer. The SOI silicon wafer was meticulously cleaned and dried, and then a 200 nm thick metal Mo layer was deposited on the top surface of the device layer as the bottom electrode by magnetron sputtering. And then, we continued to prepare 1 μm AlN as a piezoelectric layer on the wafer, and the thickness of the upper electrode prepared on the piezoelectric layer is 500 nm. The actual results will fluctuate up and down due to processing and other factors.

The piezoelectric layer was subjected to etching until the bottom electrode Mo had been completely removed. There are two main types of solutions for etching aluminum nitride: dry etching and wet etching. In this study, an alkaline solution with a higher material selection ratio and better ability to retain the metal Mo of the top electrode was chosen for wet etching the piezoelectric layer.

The top electrode was fabricated using the metal Al, which has a strong adhesion to aluminum nitride. The metal Al was patterned and bonded to the surface of the wafer. A protective layer of metal Au was then sputtered on top of the patterned Al, which helped to improve the performance and stability of the PMUT array. This layer also served as a barrier between the active material and the surrounding environment, making it more suitable for subsequent chip encapsulation.

**Figure 4:**
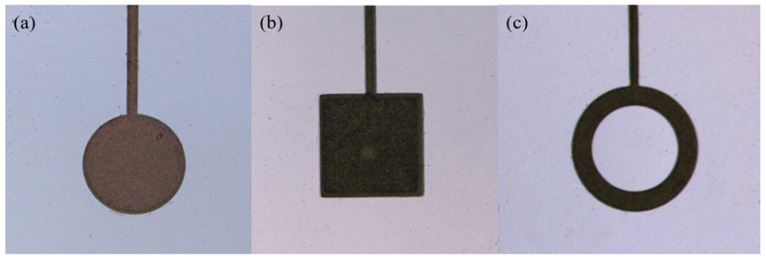
(**a**) PMUT cell with circular upper electrode. (**b**) PMUT unit with square upper electrode. (**c**) PMUT unit with doughnut upper electrode.

The back cavity etching release PMUT unit used Deep Reactive Ion Etching (DRIE) for deep silicon etching. However, long etching times can damage the original structure, so a layer of SiO_2_ was deposited on the silicon surface of the SOI bottom as a hard mask layer before deep silicon etching. This ensures that the backside cavity shape was accurately etched using Reactive Ion Etching (RIE), resulting in the complete fabrication of the PMUT cells. The fabricated PMUTs were observed under a confocal microscope, as shown in the Figure 4.

## 4. Results and Discussion

The effective electromechanical coupling coefficient ($k_{eff}^{2}$) of the PMUTs were calculated using the following Formula (3) [27]:

(3) $$k_{eff}^{2}=1-{\left(f1/f2\right)}^{2}$$

where *f*1 is the resonance peak, and *f*2 is the anti-resonance peak.

The electromechanical coupling coefficient measures the strength of the interaction between the input electric field and the output mechanical energy [28,29], which, in turn, reflects the efficiency of the transducer in converting electrical energy into mechanical energy. A higher electromechanical coupling coefficient indicates a stronger ultrasonic signal, making the PMUT highly suitable for ultrasound imaging applications.

The fabricated PMUTs were designed with three top electrode patterns and corresponding back cavity shapes. In order to maximize electromechanical coupling, the top electrodes were covered to the position where the total stress was 0, which means that the diameter of the top electrode was selected up to be 70% of the diameter of the bottom cavity [30]. The results showed that the circular patterned cell had a higher first-order resonant operating frequency, and its electromechanical coupling coefficient was calculated to be 1.43% greater than those of the other two cells.

The transmit voltage response (TVR) [25,31] is a measure of the sensitivity of a PMUT, which is defined as the ratio of the free-field acoustic pressure generated at a certain distance from the ultrasonic transducer in a specified direction to the voltage at the input of the transducer. The smaller the value of TVR is, the higher the transmit voltage response of the transducer is, i.e., the higher the sensitivity of the PMUT is. To characterize the electrical performance of the fabricated PMUT cells, COMSOL simulation was used to select the external field calculation function pext() to calculate the sound pressure of the three PMUT units operating in the first-order resonance mode at 10 mm outside the closed sound field and return the TVR value. The performance parameters of the three PMUT units were derived, as shown in Table 2.

The PMUT with a circular top electrode structure has a significantly better TVR value at 10 mm than those of the other two models. This suggests that the structure with the circular top electrode pattern and the circular back cavity is more suitable for fabricating PMUT arrays. A circular top electrode pattern and back cavity shape can help to maximize electromechanical coupling, which, in turn, leads to higher sensitivity and ultrasonic signal strength, making the PMUT more suitable for ultrasound imaging applications.

### Simulation and Fabrication of PMUT Arrays

**Figure 5:**
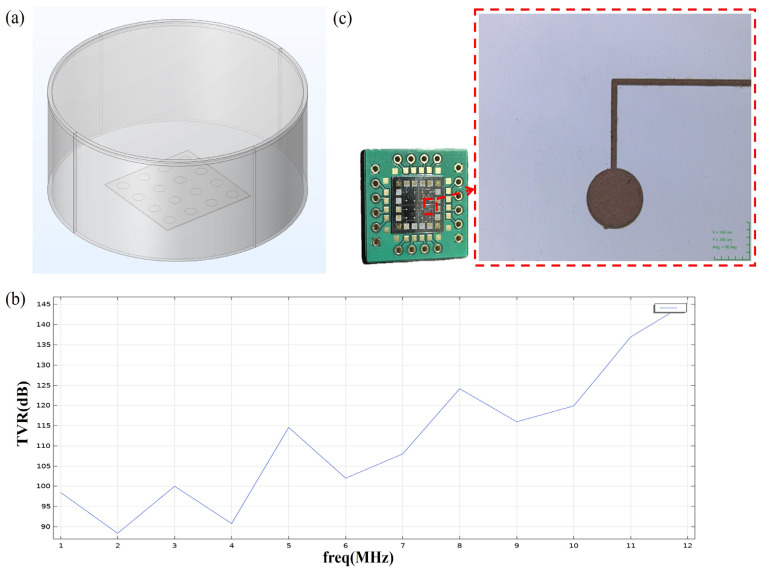
(**a**) Modelling using circular top electrode array. (**b**) TVR curve at 10 mm directly in front of the PMUT array. (**c**) Arrays manufactured similarly.

The circular PMUT array established using COMSOL is shown in the Figure 5, which is set as 4 × 4, with a total of 16 units, and the water area and the outer perfect matching layer are set right above the array. After simulation, it was found that the array operates in the frequency range of 4–6 MHz in water, and the ultrasonic signals emitted in this mode are more in line with the conventional ultrasonic frequency range for medical detection. The sound pressure response level at 10 mm directly in front of the array operating at 4–6 MHz is also simulated using external field calculations, as shown in Figure 5b.

When the array is set at 4 MHz versus a 6 MHz operating frequency, the TVR is less than the sound pressure level shown at 5 MHz. This may be due to the computational limitations of this simulation at only a few integer nodes of the operating frequency of the calculation of the TVR, but according to the graph of the trend, we observed that the TVR at 4–6 MHz first rose, and then fell. The best PMUT sensitivity should be reflected at 5 MHz or so. Figure 5c shows a photograph of a PMUT array fabricated using the typical MEMS process, including a magnified view of a single array element on the array.

## 5. Conclusions

In this study, physical and acoustic field coupling was stimulated using 3D finite element simulation accurately models, allowing for a wide range of performance parameters to be calculated for the three PMUT cells. The custom wafer SOI was used to design and fabricate the PMUT cells and arrays. The results show that the circular electrode PMUT has higher sensitivity and a higher first-order resonance frequency compared to those of the other patterns, making it more suitable for forming high-density arrays. These findings make great contributions for enhancing the signal transmission sensitivity and bandwidth of PMUTs through structure design, which have significant potential in non-destructive testing and medical imaging applications. In the future, efforts will be made to fabricate PMUTs with an even better performance for ultrasound applications.

## Figures and Tables

**Table 1:** Comparison of PZT and AlN parameters.

Property	AlN	PZT	
piezoelectricity	~−2	~−200	d_31_[pC/N]
dielectric constant	10	1500	$\varepsilon_{33}$
Poisson’s ratio	0.25	0.3	ϑ
Quality factors	~2000	50–100	Q_m_
Thermal Conductivity	170–200	1.5	W/m·K
Dielectric Permittivity	~8.5	1000–2000	
Safety	non-toxicity	toxic	

**Table 2:** Parameters corresponding to the three types of PMUTs.

	Circular	Square	Doughnut
Frequency (kHz)	513.7	509.3	507.9
TVR (dB)	74–76	116.8–117	115.8–116
$k_{eff}^{2}$	1.43%	1.26%	1.13%

## Data Availability

The data that support the findings of this study are available from the corresponding author upon reasonable request.
